# The Implication of Topoisomerase II Inhibitors in Synthetic Lethality for Cancer Therapy

**DOI:** 10.3390/ph16010094

**Published:** 2023-01-09

**Authors:** Victor M. Matias-Barrios, Xuesen Dong

**Affiliations:** 1 The Vancouver Prostate Centre, Department of Urologic Sciences, University of British Columbia, 2660 Oak Street, Vancouver, BC V6H 3Z6, Canada; 2 School of Medicine and Health Sciences, Tecnologico de Monterrey, Avenida Eugenio Garza Sada 2501, Monterrey 64849, Mexico

**Keywords:** DNA topoisomerase II, topoisomerase inhibitors, synthetic lethality, DNA damage repair, DNA repair inhibitors, Myc, EZH2

## Abstract

DNA topoisomerase II (Top2) is essential for all eukaryotic cells in the regulation of DNA topology through the generation of temporary double-strand breaks. Cancer cells acquire enhanced Top2 functions to cope with the stress generated by transcription and DNA replication during rapid cell division since cancer driver genes such as Myc and EZH2 hijack Top2 in order to realize their oncogenic transcriptomes for cell growth and tumor progression. Inhibitors of Top2 are therefore designed to target Top2 to trap it on DNA, subsequently causing protein-linked DNA breaks, a halt to the cell cycle, and ultimately cell death. Despite the effectiveness of these inhibitors, cancer cells can develop resistance to them, thereby limiting their therapeutic utility. To maximize the therapeutic potential of Top2 inhibitors, combination therapies to co-target Top2 with DNA damage repair (DDR) machinery and oncogenic pathways have been proposed to induce synthetic lethality for more thorough tumor suppression. In this review, we will discuss the mode of action of Top2 inhibitors and their potential applications in cancer treatments.

## 1. Introduction

DNA topoisomerase II (Top2) is necessary for DNA replication and transcription, as well as for chromosome condensation and segregation. Top2 inhibitors such as doxorubicin and etoposide are medications commonly used to treat breast, lung, and testicular cancer, as well as lymphomas, sarcomas, and other neoplasms, even though they present dose-limiting toxicity and side effects [1,2]. Despite the emergence of revolutionary medications in the context of personalized approaches, these therapies are still regarded as essential in cancer treatment. However, cancer cells generate resistance to these drugs by using driver genes such as Myc and EZH2 to increase Top2 catalytic function and DNA damage repair (DDR) machinery, thus overcoming replication and transcription stress and avoiding cell death. In this regard, synthetic lethality techniques may provide a broader range of druggable targets to deal with therapy resistance developed toward Top2 inhibitors, which should thus increase the susceptibility of cancer cells to genotoxic therapy. “Synthetic lethality” occurs when numerous defects in two or more related genes occur concurrently, causing cell death or apoptosis; a single loss in one of these genes that is tolerated results in cell survival [3]. With advances in genomic investigations of gene mutations and expression patterns, it is now possible to analyze the effect of single gene deletion on tumor cell survival on a large scale, allowing for the discovery of novel synthetic lethal targets. Initially, synthetic lethality strategies included combining a mutation with a drug targeting a specific tumor pathway. PARP inhibitors were the first targeted treatments to employ synthetic lethality and were used to destroying cancers through DNA repair failure (e.g., BRCA1/2 mutation) [4]. As an alternative to mutations, a second drug that functionally duplicates the effect of the mutation may be employed to generate a combination of synthetic lethality. Combination treatments based on synthetic lethality have the potential to be effective at controlling tumor growth because they permit the administration of drugs at lower dosages, reduce cytotoxicity in tumor cells, and minimize drug resistance selection. Drugs that change or impede DNA repair pathways or enhance Top2’s genotoxic effects have the potential to sensitize cells to commonly used Top2 inhibitors. They may lead to more robust tumor suppression, longer periods of remaining disease-free, increased survival, and even enhanced quality of life. 

In addition, targeting genes that potentiate the epigenetic and chromosome activity of Top2, such as Myc and EZH2, can also be used to introduce synthetic lethality. Myc has been recently used as a target for new chemotherapeutics [5,6]. Since its primary mechanism is to induce cancer survival genes [7], dual inhibition of Myc and Top2 can be beneficial. In addition, Top2 has been demonstrated to be essential in efforts to relax DNA and expose the Myc promoter region for transcription [8]. Thus, the combination of Myc–Top2 inhibition can help treat tumors. On the other hand, EZH2, which serves as an epigenetic modifier that inhibits the expression of tumor suppressors, has been shown as a potential drug target both alone and in the combination with Top2 inhibition [9]. Here, we explore the mechanism of action of Top2 inhibitors and propose the application of DDR, Myc, and EZH2 inhibitors in combination with Top2 inhibitors to induce synthetic lethality.

## 2. DNA Topoisomerases

DNA topoisomerases are well-studied proteins that are required for transcription, DNA replication, and chromosome segregation through cell cycling and act by temporarily introducing single- or double-strand breaks in DNA and then resealing them [10]. As a result, topoisomerases are essential for maintaining DNA integrity during transcription and replication when cells are proliferating.

Based on the number of DNA strands they cleave, topoisomerases can be divided into types I and II. Type I enzymes cleave just one strand of DNA, whereas type II enzymes cleave both strands to prevent supercoiling or entanglements [10,11]. Furthermore, according to the kinds of covalent phosphotyrosyl intermediate they generate (5′ or 3′ linkage), the structures and reaction mechanisms of topoisomerases can be classified into five types: IA, IB, IC, IIA, and IIB (Table 1). These enzymes function by temporarily rupturing and reuniting DNA strands. This process involves a nucleophilic assault on a DNA phosphodiester bond that is carried out by a topoisomerase tyrosine residue [12]. 

Eukaryotic type I topoisomerases include two monomeric enzymes. Type IA cleaves a single-strand segment and lets the intact strand pass through the split [13]. Topoisomerase IIIa (Top3a) and IIIb (Top3b) form the IA subfamily in humans. They are known to relax hyper-supercoiled DNA segments [14]. On the other hand, type IB allows the broken strand to revolve around the intact strand, does not need divalent metal ions, and covalently binds to the 3′-terminal phosphate of the DNA. In humans, topoisomerase I (Top1) and topoisomerase I mitochondrial (Top1mt) have been observed. They relax negative and positive supercoiled DNA. Finally, type IC works using a controlling rotation mechanism similar to the IB type; however, it is only found in *Methanopyrus genus* [14]. Although these enzymes can modulate the under- and over-winding of DNA, they cannot remove knots or tangles from duplex DNA. They also do not require a high-energy co-factor to function [15]. 

Eukaryotic type II topoisomerases, also known as Top2, are the enzymes responsible for cleaving both DNA strands and allowing the DNA duplex to continue past the breakage. These enzymes carry out their functions as homodimers and require the presence of divalent metal ions, in addition to adenosine triphosphate (ATP), for total catalytic activity [16]. The type IIB subfamily is found in the archaea and the bacteria domain of life [17], while the type IIA subfamily is found in bacteria and mammals. The topoisomerase IIa (Top2a) and topoisomerase IIb (Top2b) isoforms are found in humans. Despite their close relationship, they are encoded by two separate genes and have distinct molecular masses: Top2a is 170 kDa in size, while Top2b is 180 kDa [18]. The two enzymes have different expression patterns in vertebrate cells and thus different physiological activities. Top2a is essential to actively dividing cells, and its protein levels are regulated throughout the cell cycle, with concentrations peaking at the G2/M phase. During the process of mitosis, Top2a stays firmly attached to the chromosomes. On the other hand, Top2b demonstrates an autonomous position regarding proliferation and distances itself from chromosomes in the process of mitosis [19]. Top2b is incapable of making up for the absence of Top2a in mammalian cells, which suggests that the two enzymes serve distinct and independent functions in the body.

The functions of DNA topoisomerases are different in normal cells and cancer cells. In normal cells, they maintain genome integrity by modifying the chromatin structure and promoting safe chromatid segregation during mitosis. In cancer cells, their protein levels or activities are enhanced to cooperate with increased cell replication and to enhance cell survival by serving as a co-factor protein in biomolecular processes [20,21]. Top2a protein levels have been correlated with proliferation rate and the carcinogenesis of neoplasms in multiple types of cancers [22], and Top2a expression is associated with poor prognoses [23]. On the other hand, Top2b activity is reported to be upregulated in steroid hormone cellular signaling in breast and prostate cancers, thus aiding cancer cell survival. This survival mechanism is accomplished by an increase in double-strand breaks in androgen receptor (AR)- or estrogen receptor (ER)-positive cells, which relaxes DNA and thus allows these receptors to bind to their target promoters to induce their transcriptional activity [24,25]. These findings rationalize DNA topoisomerase inhibition as a therapeutic method. Decades of research have led to DNA topoisomerase inhibitors becoming one of the leading cancer therapeutics in the clinical setting.

## 3. DNA Topoisomerase Inhibitors

Topoisomerase inhibitors are known to be among the most powerful and most frequently prescribed anticancer treatments [26]. They target the enzymatic activities of topoisomerase in DNA cleavage and ligation processes. Inhibition of these processes will result in either increased cleavage complexes or replication stall during DNA synthesis and lead to DNA damage, cell cycling arrest, or even cell death [1]. In addition, these medications exhibit substantial selectivity and non-ambiguity by not cross-reacting to each topoisomerase [27].

### 3.1. DNA Topoisomerase II Inhibitors

**Figure 1:**
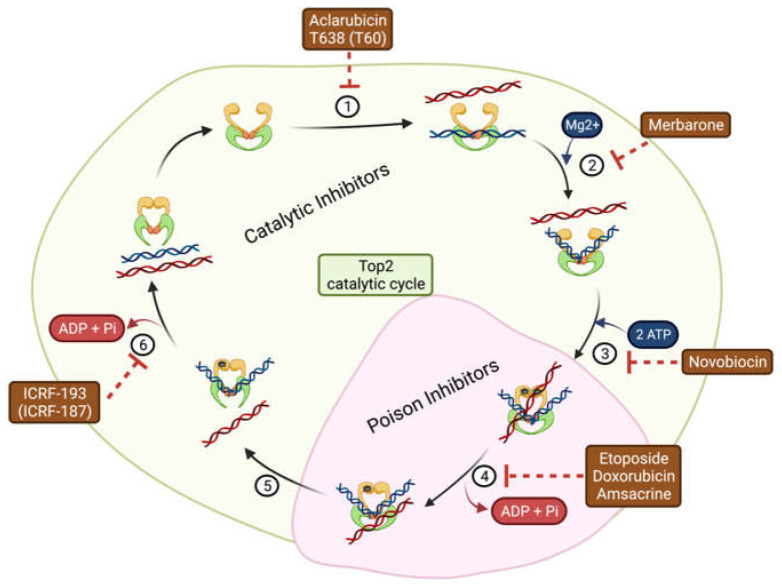
Catalytic cycle of Top2. Relaxed/tangled DNA dynamics process: (1) the enzyme binds to double-strand DNA; (2) the DNA cleavage reaction requires Mg^2+^; (3) two molecules of ATP bind to the N-terminal domain; (4) ATP hydrolysis provides the energy for DNA passage; (5) DNA repair/re-ligation; and (6) dissociation of DNA–Top2 complex after the second ATP hydrolysis. Top2 is then ready to start a new cycle of enzymatic activity. The figure was created on biorender.com.

Type II topoisomerases inhibitors block the activity of Top2a/Top2b at different stages of their catalytic cycle. The enzymes consist of three domains, namely the N-terminal domain, the cleavage/DNA binding domain, and the C-terminal domain [28]. These domains work collaboratively to create double-strand breaks. There are six steps in the Top2 catalytic cycle involving DNA structure dynamics (Figure 1): (1) Top2 binds to two DNA segments; (2) in a Mg^2+^-dependent process, one DNA strand is bent and a double-strand break is induced by initiating a nucleophilic attack on the phosphodiester DNA backbone (Tyr805 and Tyr821 in human Top2a and Top2b, respectively); (3) Top2 conformational changes following ATP binding induce segment trapping; (4) the hydrolysis of ATP facilitates the passage of the other DNA strand through the break; (5) DNA strand breakages are re-ligated; and (6) Top2 dissociates from DNA after ATP hydrolysis [29,30]. 

Top2 inhibitors contain a broad range of compounds that block Top2 activity through various modes of action. However, they can be roughly divided into two groups based on their destructive effects on cells, namely Top2 poison inhibitors and Top2 catalytic inhibitors (Table 2).

#### 3.1.1. DNA Topoisomerase II Poisons

Poison inhibitors halt the Top2 catalytic cycle following DNA cleavage. They increase the amount of Top2-DNA cleavage complexes (Top2cc), resulting in genotoxic entities within cells due to induced DNA damage caused by a deficient DNA repair system. Consequently, the buildup of these DNA breaks will eventually result in programmed cell death [41]. Etoposide, an exemplary Top2 poison, is frequently used in oncology (typically in conjunction with other chemotherapeutics) to treat a range of malignancies (including ovarian, testicular, and small cell lung cancer, as well as leukemia and lymphoma) [38]. Because of its low affinity toward free DNA, weak DNA intercalating activity, strong selectivity toward the Top2–DNA cleavage complex, and its ability to trap cleavage complexes with high frequency, etoposide is an appealing Top2 poison [42]. However, etoposide usage is associated with long-term toxicities, secondary malignancies, and dose-limiting cardiotoxicity [43]. Patients can also develop tolerance to Top2 poisons because they activate DDR machinery (phosphorylation of ATM and activation of downstream targets in both the HR and NHEJ pathways) [44]. However, a recent genome-wide investigation revealed genes that may predict resistance to Top2 poisons, increasing their potential as precision medicine therapies [45]. 

#### 3.1.2. DNA Topoisomerase II Catalytic Inhibitors

Catalytic Top2 inhibitors are chemicals that inhibit Top2 enzymatic activity. They block the enzyme before DNA breaks are carried out or after DNA re-ligation is completed. Therefore, they do not induce Top2cc accumulation. Due to the inability of Top2 to relax DNA supercoils or decatenate sister chromatids during mitosis, failed cell division and subsequent cell death can be established [46,47].

Catalytic Top2 inhibitors do not have side effects that are caused by Top2 poisons, but their therapeutic use is restricted. Despite the limited success of Top2 catalytic inhibitors in drug development, several compounds have been identified, such as novobiocin, merbarone, suramin, aclarubicin, ICRF-187, ICRF-193, T60, and T638 [32,33,34,46]. However, novobiocin, merbarone, suramin, and aclarubicin have multiple targets in addition to Top2, limiting their future characterization and development as anticancer drugs [46]. These off-target effects might be explained by poor drug development processes before clinical trials. As for T60 and its derivates, compounds designed using a novel pocket of the enzyme with computer-aided drug development methods seem to have fewer off-target effects than the known catalytic inhibitors [32]. Regarding its clinical use, ICRF-187 is authorized for human use as a calcium chelating reagent and is used to treat cardiotoxicity induced by Top2 poisons [48]. ICRF-193, T60, and T638 have demonstrated anticancer activity in animal models [32,33,49]. While several Top2 catalytic inhibitors are undergoing preclinical development and have advanced to varying stages of research [32,50,51], Top2 catalytic inhibitors offer promise as potential anticancer medicines.

## 4. Double-Strand Break (DSB) Repair

DNA DSBs can be lethal to cells. They require immediate repair to avert potentially harmful chromosomal disruption. On the DNA lesion site, several DSB sensors are needed to detect DNA damage and activate downstream effectors. PARP1 and the Ku70/Ku80 complex are the first proteins recruited to DNA DSBs [52]. They target proteins to promote chromatin de-condensation and recruit successive second-cell messengers. 

Once the lesion has been identified, ATM is recruited by the MRN complex (RAD50, NBS1, and MRE11) to create a checkpoint arrest amplification. Additionally, ATM activates the histone H2AX, which results in the production of gamma-H2AX (H2AX) [53]. This modification is necessary for the recruitment of several factors, such as the DNA damage checkpoint 1 mediator (MDC1), and it is accompanied by a concomitant increase in the E3 ubiquitin–protein ligase RNF168, which enhances chromatin relaxation even further and makes it possible to recruit additional DNA repair factors [54]. 

**Figure 2:**
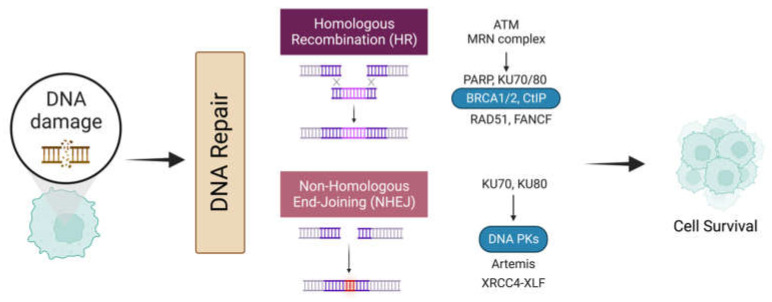
DNA damage response and repair pathways. Several DSB repair mechanisms are available based on the phase of the cell cycle and the existence of homology sequences in the DNA strands. The DNA repair mechanism could undergo the HR or the NHEJ pathway according to the cell cycle stage at the moment of the lesion and the acquired mutations in the cell. The figure was created on biorender.com.

During this DSB recognition stage, the decision of applying homologous recombination (HR) or a non-homologous end-joining (NHEJ) repair mechanism depends mainly on the cell cycle phase during DNA resection (Figure 2). DSB end resection stimulators favor HR at the S and G2 phases, primarily through CtIP and BRCA1 and MRN complex [55]. On the other hand, during the G1 phase of the cell cycle, the DSB end resection process is inhibited by the Ku70/Ku80 heterodimer and TP53BP1 [56].

### 4.1. DNA Repair Mechanism

DNA repair is regulated by a complex network of sensors and effectors and begins with the identification of DNA damage and the selection of the most viable repair route, which is determined by the cell cycle stage and the type of DNA damage. During the G0/G1 phase, small lesions are resolved by nucleotide excision repair and base excision repair. During the S phase, DNA is repaired using the Fanconi anemia. mismatch repair DSBs, Alt-NHEJ (alternative non-homologous end-joining), and single-strand annealing. Each of these repair mechanisms is unique [57]. However, they are not mutually exclusive, which creates a network of proteins that share DNA repair functions. Mutations in these systems may cause an accumulation of poorly repaired DNA, predisposing cells to cancer [58]. 

Additionally, abnormalities in DNA repair systems that preserve genome integrity allow cancer cells to become aggressive and resistant to cancer therapy [59]. These abnormalities in cancer cells include overexpression, downregulation, mutations, and the polymorphism of proteins in different DNA repair pathways. In the NHEJ pathway, overexpression of the Ku70/80 complex has been identified in gastric and breast cancers [60,61]. DNA-PK was reported to be highly expressed in oral, lung, and esophageal carcinoma [62]. As for the HR pathway, BRCA1/BRCA2-deficient cells are found in breast cancers [63]. However, overexpression of RAD51 genes has also been demonstrated in pancreatic cancer and leukemia [64]. Thus, cancer cells can optimize cell survival by enhancing their intact DNA repair mechanism in environments where the other pathway is defective. Thus, this generates an opportunity to treat cancer cells with synthetic lethality by inhibiting the remaining intact DNA repair pathway and other DNA damage agents.

#### 4.1.1. Homologous Recombination

MRE11’s endonuclease activity cleaves the DNA close to the DSB in HR and then removes the DNA edges [65]. The replication protein (RPA) will then bind the DNA after forming a single strand to prevent it from being degraded [66]. RPA is then displaced by BRCA2 and PALB2 to increase the binding of RAD52 to ssDNA [67]. This mechanism is believed to displace the Ku70/Ku80 complex in order to allow resection factors, including DNA2, EXO1, BLM, WRN, and RPA, to approach the target sites [68]. 

Subsequently, RAD41 invades the complementary strands containing homologous sequences, while RAD51 mediates complementary annealing of the DNA strands by mediating the DNA–DNA interaction [69]. Subsequently, a displacement loop (D-loop) is generated by lengthening the DNA strand through the use of replicative or translation DNA polymerases [70]. At this point, three different pathways can be used to resolve D-loops. Double-strand break repair (DSBR) induces the production of crossover and non-crossover products [71]. Synthesis-dependent strand annealing (SDSA) then increases the non-crossover effects [72]. Finally, break-induced replication (BIR) generates half-crossover products and frequently promotes mutagenesis [73]. It has been shown that TP53BP1 promotes a fork cleavage-free pathway, while BRCA1 facilitates the BIR pathway coupled with SLX–MUS complex-mediated fork cleavage [56]. This TP53BP1 function is performed by reducing the activity of helicases, which limits D-loop stability and makes it more likely for crossover and BIR events to occur.

A systematic review of 33 types of cancer revealed that multiple mutations in the HR pathway create a defected DNA repair mechanism in ovarian cancers. The most common mutations in the HR pathway occur in the BRCA1, BRCA2, RAD51, BLM, and RAD50 genes [74]. RAD51 overexpression has been linked to worse prognosis in patients with sporadic gastric cancer and neuroblastoma who present modified MRE11 [75,76]. In contrast, RAD50 and NBS1 mutations are associated with carcinogenesis during endometrial and prostate cancer development [77,78].

#### 4.1.2. Non-Homologous End-Joining

NHEJ is the principal mechanism by which DSBs are repaired in human cells. During the classical NHEJ (c-NHEJ) pathway, Ku70/Ku80 heterodimer binds to the DSB ends before resection, allowing for the recruitment of DNA-PK. The endonuclease ARTEMIS then processes the fragmented ends until cohesion is restored. In the final step, DSB ligation is accelerated by DNA ligase 4 interacting with NHEJ factor 1 (XLF) [79]. Alternatively, DSBs may be repaired by SSA or Alt-NHEJ, which leads to DNA deletion depending on the extent of end resection. These pathways require ATM signaling [80]. In the SSA pathway, RAD52 anneals homologous sequences during resection, while DNA polymerase fills gaps [81]. On the other hand, during MMEJ, PARP1 works together with helicases to displace RPA from ssDNA. This process reveals microhomologies and promotes the stabilization of the ssDNA ends [82]. To finalize the ligation process, DNA ligase 3 (LIG3) forms a complex with X-ray repair cross-complementing protein 1 (XRCC1) to catalyze the reaction [83]. The decision between c-NHEJ and Alt-NHEJ is influenced by the WRN complex, which prevents MRE11 and CtIP from being recruited on the DNA lesion, thus favoring c-NHEJ. Another critical step in Alt-NHEJ and SSA is the elimination of non-homologous DNA strands, which is mainly mediated by nucleases [84]. 

One of the mechanisms of PARP1i resistance during this DNA repair pathway is the decreased expression of proteins in the ATM–CHK2 pathway. This process has been linked to the downregulation of the late-phase HR factor TP53BP1 [85]. In general, abnormal expression of the TP53BP1 protein has been shown to contribute to tumor growth [86,87]. 

In addition, relatively few malignancies have been linked to the downregulation or modification of genes involved in c-NHEJ. In colon and endometrial cancers, only uncommon Ku70/Ku80, LIG4, ARTEMIS, and XLF mutations were identified [88]. 

Alt-NHEJ and SSA are innately mutagenic due to the production of deletions and the creation of genomic instability, which is associated with many neoplasms. Other genes have been postulated to be potentially carcinogenic through routes such as CtIP [89]. CtIP inactivation reduced carcinogenesis in a mammary mouse model lacking TP53 [90]. In addition, Alt-NHEJ is dependent on Polθ, and enhanced POLQ protein expression has been reported in various cancers, including breast and ovarian cancer [91].

## 5. Topoisomerase II inhibition Synergizes with DNA Repair Inhibitors

**Figure 3:**
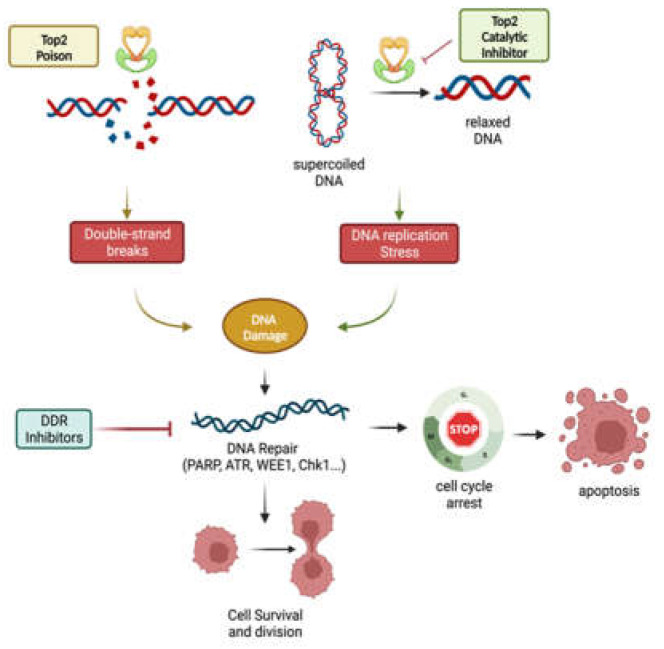
Synthetic lethality of Top2 inhibition in combination with DDR inhibitors. Top2 inhibition generates DNA damage during double-strand breaks or DNA replication stress. These perturbations to the cell can be abolished by DNA damage repair pathways, thus allowing the cell to survive. However, inhibiting the proteins involved in the repair mechanism can increase the efficacy of Top2 inhibitors by producing synthetic lethality. The figure was created on biorender.com.

The key role of DNA repair pathways in cancer cells when encountering DNA damage created by Top2 poisons inhibitors rationalizes therapies targeting the remaining functioning pathways that are essential for cancer cell survival and proliferation. This is the foundation of the notion of synthetic lethality, which is the genetic interaction of two genes. Cell viability is unaffected if only one of the two genes is altered. However, cell death ensues when both genes are mutated simultaneously. Identifying several synthetic lethal interactions between proteins involved in DNA repair enabled the creation of tailored therapeutic combinations that target DNA repair enzymes and topoisomerase II inhibitors with the aim of eradicating cancer cells. This dual inhibition ensures that malignant cells are forced into apoptosis by mitosis catastrophe without trying to repair the damage caused by cell cycle arrest (Figure 3). Numerous DDR-targeting compounds are being investigated in clinical trials, with encouraging outcomes for cancer therapy expected (Table 3).

### 5.1. PARP Inhibitors

Poly (ADP-ribose) polymerase-1 inhibitors (PARP1i) are the first effective instance of targeted medicines that employ synthetic lethality to eradicate malignancies through DNA repair failure [4]. They were first utilized in cancers with BRCA1/BRCA2 mutations. Olaparib was the first FDA approved inhibitor of PARP1, and several derivates, along with new molecules, are now being investigated for treating BRCA-mutant cancer [4,104,105]. Due to PARP1’s functions in DNA repair, replication stress responses, and chromatin remodeling, other applications for PARP1i in combination treatment are still being investigated [106]. Overexpression of PARP1 and other DNA damage repair proteins may aid in repairing DNA lesions caused by Top2 inhibitors, allowing tumor cells to withstand treatment involving Top2 inhibitors [107,108]. Thus, Top2 and PARP1 dual inhibitions are considered an alternative for achieving a synergistic impact on tumor cells. BRCA1 maintains genome integrity by preventing the formation of estrogen-induced pathogenic Top2–DNA complexes [109]. BRCA1-deficient cells exhibited hypersensitivity to etoposide and daunomycin that was comparable to Olaparib [110]. A recent study shows that in ovarian cancer cells, the combination treatment of the topoisomerase inhibitor and PARP inhibitor (PARPi) is superior to PARP inhibition or topoisomerase inhibition alone [107].

### 5.2. ATM/ATR Inhibitors

ATM and ATR activate DDR repair pathways for cell survival in response to DNA damage. Thus, they play a crucial role in cancer treatment. The ATR protein is localized to the DSB site by the ATR-interacting protein (ATRIP) after RPA coats the ssDNA. Consequently, the CHK1 signaling cascade will induce G2-M phase cell cycle arrest, allowing DNA damage to be fixed. On the other hand, ATM uses the MRN complex to respond to DNA damage [44]. By inhibiting these major proteins (ATR and ATM), DNA damage response pathways can be interfered with, causing unresolved DNA damage in growing cancer cells and, eventually, cell death. Non-tumor cancer cells may endure DNA damage because they possess additional DDR components, such as MMR and NER, that detect and fix DNA replication mistakes. As a result, this inhibition may be a successful method in cancer therapy. Some small molecules of ATM and ATR inhibitors have been developed and are being investigated in preclinical trials (Table 3).

Since ATM and ATR are the first molecules activated after DNA damage, their dual inhibition with Top2 inhibitors made them a potential combination therapy for cancer proliferation arrest and apoptosis. In a recent study, ATM inhibition increased the susceptibility of tumor cells to Top1/Top2 inhibitors (e.g., camptothecin, doxorubicin) [111]. In addition, ATR downregulation also increased the efficacy of topoisomerase poisons [112].

### 5.3. WEE1/CHK1 Inhibitors

CHK1 and WEE1 are overexpressed in various tumors [113,114,115]. The WEE1 kinase halts the cell cycle at the G2/M phase by inhibiting CDK2/CDK1, which is a process that allows DNA repair mechanisms to be carried out [116]. Inhibiting WEE1 will cause tumor cells to become more susceptible to DNA damage treatments. The CHK1 kinase has several roles in DNA damage-activated signal pathways. CHEK1 inhibition can cause cell cycle arrest at the G2 and S checkpoints, promote DNA damage accumulation, and eventually lead to tumor cell death. Through suppressing CHK1, CHK2, or WEE1, cells suffering DNA damage would lack the ability to activate the checkpoint mechanism and therefore fail to arrest the cell cycle resulting in mitosis aberration with the proportional accumulation of lethal mutations to the cells [117]. This uninterrupted cell replication eventually leads to cell death. 

Different approaches have been created to test synergism between Top2 and WEE1/CHK1/CHK2 inhibitors. Interestingly, CHK1 and Plk1 regulate mitotic entrance after Top2 catalytic inhibition, which opens the possibility that its inhibition will lead to a mitotic catastrophe instead of cell cycle arrest [118]. WEE1 inhibition makes ovarian, colon, cervical, osteosarcoma, glioblastoma, and lung cancer cells more vulnerable to DNA damage caused by irradiation and topoisomerase inhibition [119]. In addition, the apoptotic effects of doxorubicin were greatly enhanced in all cell lines by suppressing the expression of WEE1 [120].

### 5.4. DNA-PK Inhibitors

DNA-PK is a crucial response to DSBs in the c-NHEJ pathway. c-NHEJ is initiated by the Ku complex. DNA-PK then binds to the DSB and begins DNA repair [121]. It has been discovered that overexpression of DNA-PK in tumor tissues after radiation therapy and DNA-PK inhibition decreases DNA repair, indicating that DNA-PK plays a role in overcoming the drug resistance of tumor cells and that DNA-PK inhibition has a superior therapeutic impact. As a result, DNA-PK has been proposed as a viable pharmacological target for anticancer therapies [122,123,124].

Current studies that combine peposertib (a DNA-PK inhibitor) with Top2 inhibitors (e.g., doxorubicin and etoposide) showed the improved effectiveness of these drugs in ovarian cancer xenografts [125]. Compared to monotherapy controls, the combination of peposertib and Top2 inhibitors reduced tumor development in murine grafts. A clinical study of peposertib in conjunction with cytotoxic treatment is now underway for ovarian cancer [126]. In addition, new DNA-PK inhibitors such as ZL-2201 have demonstrated notable synergy with Top2 inhibitors regardless of ATM status [127]. Moreover, the discovery of a need for TP53 in Top2a-dependent G2 arrest and the activation of DNA-PK pathways opens the possibility of inducing selective synthetic lethality by blocking either PK or the TP53 protein [128].

## 6. Chromatin Remodeling and Gene Transcription

Restoring damaged DNA requires chromatin remodeling, which involves epigenetic modifications to histones (e.g., methylation or acetylation) [129]. These modifications may activate or inhibit gene clusters responsible for cancer cell survival [130]. In addition to histone modifiers, DNA topoisomerases are eukaryotes’ most essential chromatin dynamic regulators. Top2 recruitment affects the epigenetic state of target promoters regulated by histone modifiers, thus resulting in chromatin accessibility [131]. Indeed, the loss of Top2a activity is associated with histone modifications, as seen in decreased H3K27me3 enrichment [132,133]. Besides Top2’s functions as a chromatin modulator, epigenetic modifier, and DNA damage inducer, it also plays a role in inducing gene transcription [12]. This transcriptional mechanism is induced by DSBs in gene promoter sites [12,134,135].

During neuronal differentiation, Top2b induces housekeeping genes while proliferation genes are silenced. Additionally, Top2a influences development-specific genes in embryonic stem cells targeting bivalent histone modifications, and these genes are expressed and occupied by Top2b during differentiation [136]. This provides a rationale for combining Top2 inhibitors with inhibitors of histone modifiers or cancer cell survival genes to create a novel synthetic lethality approach for cancer therapy.

### 6.1. EZH2 and Topoisomerase II

Enhancer of zeste homolog 2 (EZH2) is a member of the polycomb group of genes, which is a family of essential transcription-repressing epigenetic regulators. EZH2 is a component of polycomb repressive complex 2 (PRC2) that methylates the Lys-27 residue in histone 3 (H3K27me3) to repress gene transcription [9]. EZH2 controls the trimethylation of H3K27 and is overexpressed in kidney, breast, and lung cancers, increasing cell motility, colony formation, and genomic instability [137]. The significance of its involvement in cancer pathophysiology is generally recognized by its functions in cell proliferation, apoptosis, and senescence. Therefore, targeting EZH2 for cancer treatment is now a popular area of research, and several EZH2 inhibitors have been developed [9]. Tazemetostat is an EZH2 inhibitor that prevents H3K27 methylation. By promoting the buildup of excessive DNA damage, tazemetostat sensitizes cells to genotoxic treatments (such as the use of Top2 inhibitors), resulting in their death [138,139]. 

Recent investigations have demonstrated EZH2 overexpression in metastatic prostate cancers [140,141,142,143]. Its overexpression has been especially linked to neuroendocrine prostate cancer (NEPC) progression. EZH2 was reported to suppress AR signaling to induce the development of neuroendocrine prostate cancer by forming the N-Myc/AR/EZH2-PRC2 complex. This gene suppressor mechanism is particularly reliant on the presence of EZH2 [142,144]. In prostate cancers, EZH2 has dual functions in that it acts as a transcriptional activator mediated by the induction of AR-regulated genes and as a gene silencer mediated by the epigenetic modification suppression of AR-regulated genes [145]. In AR-negative NEPC cells, enzymatic EZH2 inhibitors are substantially more effective than their counterparts since their actions in AR function in prostate adenocarcinoma are independent of their catalytic activity. Compounds capable of degrading EZH2 protein, analogous to EZH2 knockdown, could outperform enzymatic EZH2 inhibitors significantly and would have superior specificity in inhibiting both of EZH2’s activities [146].

Top2 inhibitors can play a critical role in the synergism of EZH2 inhibitors. By promoting chromatin relaxation and accessibility, EZH2 inhibitors create the optimal scenario for Top2 poisons to be able to access DNA double strands and generate breaks [147]. Increased accessibility to the H3K27me3-marked chromatin in leukemia cells after treatment with EZH2 inhibitors suggests that Top2 inhibitors induce DNA damage and cell death [148]. Additionally, the use of doxorubicin with EZH2 inhibitors increases the production of proapoptotic genes, which may have contributed to the death of AML cells [9]. In preclinical prostate cancer mouse cell line models, the simultaneous elevation of Top2a and EZH2 results in hypersensitivity to combined treatment with etoposide, a Top2-targeting toxin, and inhibitors of EZH2 [149]. In addition, combined EZH2 and Top2 inhibition is an alternative treatment for EGFR-mutant tumors, including those that continuously acquire resistance to EGFR tyrosine kinase inhibitors [150].

Furthermore, Top2 catalytic inhibitors can be combined with EZH2 degraders to abrogate AR transcriptional activity, which is methylation independent. Top2 increases androgen signaling and promotes tumor cell growth by relaxing chromatin, exposing promoter regions, and enhancing the transcription of androgen- and estrogen-responsive genes [24]. Catalytic Top2 inhibitors disrupt AR signaling and reduce tumor formation in castration-resistant xenografts, suggesting their potential application in the treatment of castration-resistant cancers [49]. Furthermore, the use of catalytic Top2 inhibitors such as ICRF-193 or T60/T633 in combination with enzalutamide has demonstrated a synergistic effect that blocks AR signaling and cancer cell replication [32,33,34,49]. In addition to the AR activity abolishment created by Top2 catalytic inhibitors in PCa genesis, they might also be used to reduce stem gene transcription by inhibiting Top2, as previously demonstrated by ICRF-193 in neuronal ES cells reducing essential genes such as LIN28 and NANOG with the extra function of Top2 inhibitors, thus inducing cell cycle arrest [136]. This stemness gene suppression suggests the possibility of using a continuum of Top2 catalytic inhibitors in the progression of PCa disease with the synergism of an EZH2 degrader in AR-dependent PCa cells and EZH2 catalytic inhibitors in NEPC tumor cells.

### 6.2. Myc and Topoisomerase II

The Myc protein family regulates oncogenic gene networks inside cancer cells through transcriptional programming to promote cell proliferation, metabolism, anti-apoptosis, and the inhibition of differentiation [7,151]. Dysregulation of Myc, particularly N-Myc, is correlated with the development of therapy-resistant tumors [7,152]. 

L-Myc, c-Myc, and N-Myc are involved in the development and progression of the whole spectrum of prostate cancer, from localized adenocarcinoma to the most advanced and treatment-resistant forms, including castration-resistant prostate cancer (CRPC) and neuroendocrine prostate cancer (NEPC) [153,154]. The most detected genomic changes linked to clinical phases and subtypes of PCa are amplifications of Myc family members. L-Myc amplification is mutually exclusive with c-Myc in 27% of localized PCa, while c-Myc amplification is prevalent in all PCa stages and subtypes [155]. Notably, c-Myc overexpression inhibits the transcriptional activity of the androgen receptor (AR), which is the driving force in PCa and the principal therapeutic target in the advanced tumor stage [156]. c-Myc overexpression increases AR-V7, the constitutively active and ligand-independent AR splice variant that promotes CRPC [157]. N-Myc amplification is significantly upregulated in NEPC [158]. N-Myc increases the stability of AURKA by blocking its association with the E3 ubiquitin ligase FBXW7 and binds to the promoters of target genes such as NSE, Syn, and AR to control their expression, ultimately resulting in NEPC tumor progression [159]. In addition, the development of NEPC is facilitated by the synergistic stimulation of the Wnt/β-catenin signaling pathway, which is enabled by amplifying the ALK and N-Myc genes [153].

Myc transcriptional activity is associated with Top2, whereby Myc puts together a complex called the “topoisome” to open chromatin and access promoter regions [8]. This complex is made up of Top1/Top2 proteins [8]. c-Myc joins with Top1 and Top2a in proliferating cells, while N-Myc only joins with Top1 and Top2b in terminal differentiating cells. This differentiation in topoisome proteins is in line with the fact that N-Myc is highly expressed in post-mitotic cells such as neurons. The topoisome controls chromatin morphology to maintain high transcriptional output and makes it possible for Myc to transcriptionally regulate cancer cell proliferation [8]. 

**Figure 4:**
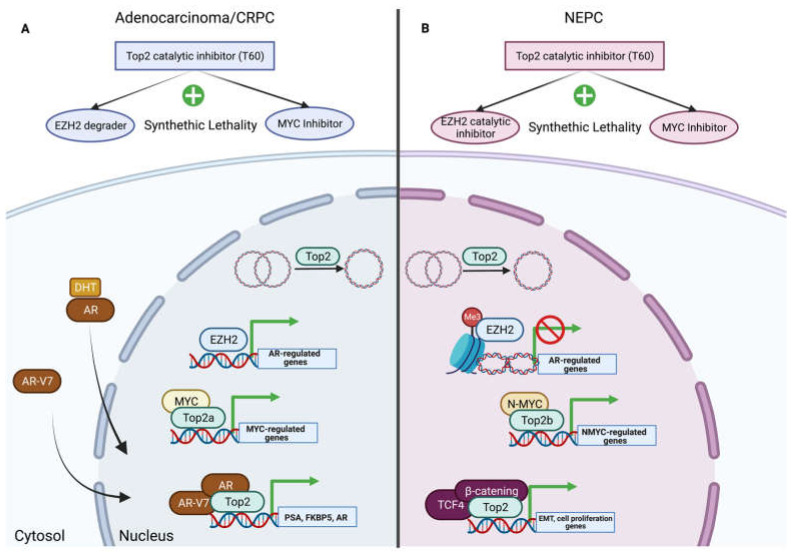
Top2 inhibition synthetic lethality with Myc/EZH2. (**A**) Top2–EZH2–Myc relationship with AR-dependent and CRPC tumor cell survival signaling pathways. (**B**) Top2–EZH2–Myc relationship with NEPC survival signaling pathways. The figure was created on biorender.com.

The discovery of new compounds that target N-Myc offers considerable therapeutic promise for NEPC. Recent research has discovered new ways of targeting Myc proteins by breaking up the Myc–Max heterodimer or stopping the Myc–Max–DNA complex from forming [5,6,160]. Top2 catalytic activity, which is required for chromatin remodeling and accessibility, should also be considered a significant molecule in Myc function. It can also be targeted and provides a proof-of-principal for the development of synthetic lethality strategies that use both N-Myc and Top2 catalytic inhibitors (Figure 4).

Dual Myc–Top2 catalytic inhibitors can be used in different phases of PCa development with a particular mechanism of action that is different for each survival pathway at different tumor stages. In CRPC, where c-Myc is most abundant, AR-V7 or AR splicing variant signals can be abolished by the dual inhibition of Top2 and Myc [32,49]. On the other hand, during NEPC development, Top2 catalytic inhibitors will prevent DNA accessibility for N-Myc, leading to survival transcription gene deficiency and cell death. Besides direct gene transcription arrest, the Top2 catalytic inhibitor abrogates the Wnt-β-catenin epithelial to mesenchymal transition pathway, which is also regulated by N-Myc [161]. Top2a physically interacts with β-catenin and enhances β-catenin’s entrance into glioma cell nuclei [162]. Top2a substantially increases the metastatic behaviors of glioma cells in a β-catenin-dependent way. Top2 inhibitors block the transcriptional activity of the TCF/lymphoid enhancer factor mediated by β-catenin [163]. Together, these findings underscore the potential application of therapies that co-target Top2 and Myc to achieve complete tumor suppression. 

## 7. Top2 Catalytic Inhibitors and Synthetic Lethality

DNA repair is a typical process that occurs in cells after DNA has been damaged. Therefore, activity can be boosted by blocking DNA repair proteins such as PARP1 or ATM/ATR. This creates the opportunity to co-target the DNA repair mechanism and Top2 activity. Therapies with catalytic Top2 inhibitors will significantly slow the pace of cancer cell replication, decrease Top2 mobility, and prevent Top2 recruitment to DSBs and gene promoters [46]. Thus, Top2 catalytic inhibition is an underestimated approach in the treatment of tumor cells. Several Top2 catalytic inhibitors have been used for decades to stop cell replication without their mechanism of action on chromatin dynamics and transcription regulation having been explored. Although ICRF-193 is a compound under clinical trials for adjuvant treatment for some cancers, it is far from becoming a key regulator in cancer treatment. Deficiencies in drug development techniques and the use of previous toxic non-selective skeleton molecules make this kind of drug unsuitable for further development. DNA–protein interactions (such as those found in Myc–Max inhibitors) and protein degradation (such as that caused by EZH2 inhibitors) have been proposed as novel therapeutic means to target critical components in cancer survival pathways [5,6,9]. Although some previous drugs have emerged to prevent Top2–DNA interaction pathways, such as aclarubicin, their mechanism of action is not entirely elucidated [46]. This prevents them from advancing to clinical trials.

Our earlier research used a computer-aided drug design approach to discover a druggable site at the Top2–DNA interaction interface. We also designed a series of small molecule inhibitors to prevent Top2 from binding its DNA substrate. Both methods were successful. The lead compound, known as T60, has demonstrated highly effective inhibitory effects against both Top2a and Top2b. Despite its low genotoxicity, it suppresses the multiplication of cancer cells. Importantly, T60 and its derivative T638 have also been shown to block the activity of the AR and the proliferation of AR-positive prostate cancer cells. These findings demonstrate that it is a viable pharmacological candidate that warrants further investigation [32,33,34]. Because it has low cytotoxicity toward cells, T638 can be combined with other cancer drugs to increase the level of tumor suppression with minimum doses, making it a suitable candidate for combination therapy with DNA repair inhibitors without the side effects associated with high doses of Top2 poisons.

## 8. Conclusions

Several benefits are associated with synthetic lethality-based therapeutic techniques, such as the ability to overcome resistance to current targeted cancer therapies and the capacity to provide synergism when paired with other chemotherapies. Several DDR-targeting drugs are now being tested in clinical trials and can be further investigated in combination with Top2 inhibitors to increase tumor treatment sensitivity by preventing cancer cell DNA repair. Although Top2 inhibitors are among the oldest target treatments, there are still unknown molecular mechanisms involved in cellular reactions to Top2 inhibitors that might enable novel synthetic lethality medicines, such as the synergism between chromatin remodeling and transcription pathways that can be observed in Myc and EZH2 signaling. Catalytic Top2 inhibitors may play a crucial role in combination therapy since large doses of these drugs may be employed to inhibit Top2 activity without causing significant genotoxicity. In addition, Top2–DNA interaction disruptors can be used to target cell proliferation in ES cells and inhibit the transcription of neurological factors in neuroendocrine or post-mitotic cells. This form of Top2 inhibition is poorly investigated but has the potential to be used in co-targeting therapies with other tumor-surviving genes. 

## Figures and Tables

**Table 1:** Type and function of topoisomerases.

Type	Subfamily	Mechanism	Human Proteins	Function	Co-Factor	Substrate
I	IA	Strand passage	Top3a	Relaxed DNA Sc, decatenation activity	Mg^2+^	DNA Sc, hemicatenanes, double Holliday junctions, and D loops.
			Top3b	Relaxed DNA Sc, RNA helicase activity		DNA HSc, RNA knots, and R loops.
	IB	Controlled rotation	Top1	Relaxed negative and positive DNA Sc	None	DNA positive and negative Sc.
			Top1mt			
	IC		Not found in humans			
II	IIA	Strand passage	Top2a	Relaxed negative and positive DNA Sc, potent decatenation activity	Mg^2+^, ATP	DNA positive and negative Sc, DNA knots, and DNA catenanes.
			Top2b			
	IIB		Not found in humans			

Sc: supercoiled; HSc: hyper-supercoiled; mt: mitochondrial.

**Table 2:** Topoisomerase II inhibitors.

Name	Mechanism of Action	Mode of Inhibition	Application	Refs.
Aclarubicin	Prevents binding of Top2 to DNA	Catalytic	Acute myeloid leukemia	[31]
T638 (T60)			Inhibition compared to etoposide with less cytotoxicity in K562 cancer cells and xenografts	[32,33,34]
Novobiocin	Binds to ATP binding site		BRCA-deficient tumors with acquired PARP inhibitor resistance	[35]
Merbarone	Blocks DNA Cleavage		Limitations due to nephrotoxicity	[36]
ICRF-193 (ICRF-187)	Blocks ATP hydrolysis and traps enzymes in the closed clamp		Addresses cardiotoxicity caused by Top2 poison	[37]
Etoposide	Stabilizes covalent cleavage complexes	Poison	Small cell lung cancer, lymphomas, refractory testicular tumors	[38]
Doxorubicin			Leukemia, ovarian and breast carcinomas	[39]
Amsacrine			Acute myeloid leukemia	[40]

**Table 3:** DNA damage repair inhibitors.

DNA Repair Protein	Inhibitor	Application	Clinical Trial	Refs.
**PARP**	Olaparib	BRCA or HR+ ovarian cancer mutations	NCT02476968 NCT03286842	[92,93]
**ATM**	AZD0156	Advanced solid tumors	NCT02588105	[94]
	AZD1390	Non-small cell lung cancer and brain cancer	NCT03423628 NCT04550104	[95,96]
**ATR**	M6620	Metastatic urothelial cancer and solid tumors	NCT02589522 NCT02567409	[97,98]
**CHK1**	AZD7762	Refractory solid tumors	NCT00937664	[99]
**WEE1**	AZD1775	Glioblastoma and refractory solid tumors	NCT02095132 NCT01849146	[100,101]
**DNA-PK**	VX-984	Advanced solid tumors	NCT02644278	[102]
	M3814	Advanced rectal cancer	NCT03770689	[103]

## Data Availability

Not applicable.
